# Analysis of tumor-infiltrating lymphocytes as prognostic factor in triple-negative breast cancer: A protocol of systematic review and meta-analysis

**DOI:** 10.1371/journal.pone.0342085

**Published:** 2026-05-12

**Authors:** Luciana Carla Martins de Aquino, Elisandra Inara Silva Andrade, Luana Maria Ferreira Nunes, Edilmar de Moura Santos, Ayane Cristine Alves Sarmento, Kleyton Santos de Medeiros, Ana Katherine Gonçalves

**Affiliations:** 1 Oncology Department, Liga Contra o Câncer, Natal, Rio Grande do Norte, Brazil; 2 Department of Medicine, Federal University of Rio Grande do Norte (UFRN), Natal, Rio Grande do Norte, Brazil; 3 Radiotherapy Department, Liga Contra o Câncer, Natal, Rio Grande do Norte, Brazil; 4 Institute of Education, Research and Innovation, Liga Contra o Câncer, Natal, Rio Grande do Norte, Brazil; 5 Postgraduate Program in Health Sciences, Federal University of Rio Grande do Norte (UFRN), Natal, Brazil; 6 Department of Obstetrics and Gynecology, Federal University of Rio Grande do Norte (UFRN), Natal, Brazil; Qatar Biomedical Research Institute, QATAR

## Abstract

**Purpose:**

Breast cancer is the most common malignant tumor among women worldwide, with an estimated 2.3 million new cases and nearly 670,000 deaths reported in 2022. The absence of hormone receptors and HER2 in triple-negative breast cancer (TNBC) reduces therapeutic options, contributing to a poorer prognosis. Tumor-infiltrating lymphocytes (TILs) are cells present in the tumor microenvironment, associated with a better prognosis and potentially playing a role in patient stratification and therapeutic management in the future. This protocol aims to assess the prognostic value of TILs in the survival of patients with TNBC, as well as their relationship with disease recurrence.

**Methods:**

Comprehensive search will be conducted in the following databases: PubMed, Embase, Lilacs, Web of Science, Scopus, SciELO, ScienceDirect, and the Cochrane Library. The search will include cohort, case-control, and clinical trials studies that approach the prognostic value of TILs in TNBC and their role in optimizing treatment strategies, and no language restrictions. Two independent reviewers will select articles based on predefined inclusion and exclusion criteria, extract data, and assess the risk of bias using the QUIPS tool. Data synthesis will be conducted using the “meta” package in R software, version 4.3.1.

**Conclusions:**

This systematic review will synthesize current evidence on the prognostic significance of TILs in triple-negative breast cancer and their association with patient survival and disease recurrence. The findings may contribute to a better understanding of TILs as potential biomarkers for prognosis and guide future therapeutic strategies in TNBC management.

**Review registration:** CRD42024568600.

## Introduction

Breast cancer is the most frequently diagnosed malignant tumor among women worldwide. Despite significant advancements in early detection and treatment strategies, it continues to be associated with high mortality rates [1,2]. According to the most recent global estimates from GLOBOCAN 2022, approximately 2.3 million new cases of breast cancer and nearly 670,000 deaths were recorded worldwide, making breast cancer the leading cause of cancer-related mortality among women [3–8].

This disease is biologically complex and heterogeneous, characterized by diverse somatic alterations that contribute to variable clinical behavior [9]. Accordingly, breast cancer is classified into different molecular and histological subtypes based on these biological features [2]. From a molecular standpoint, the main subtypes include estrogen receptor (ER)-positive, progesterone receptor (PR)-positive, human epidermal growth factor receptor 2 (HER2)-positive, and triple-negative breast cancer (TNBC). The latter is defined by the absence of ER, PR, and HER2 expression [10].

The lack of these molecular targets in TNBC significantly limits therapeutic options [11], posing challenges for both diagnosis and treatment [12]. Consequently, TNBC is regarded as the most aggressive breast cancer subtype, accounting for approximately 15% to 20% of all invasive breast cancers [12–15].

Importantly, TNBC is not a single entity but rather a highly heterogeneous disease comprising multiple molecular subtypes, such as basal-like, mesenchymal, immunomodulatory, and luminal androgen receptor (LAR) subgroups, each with distinct prognostic features and potential therapeutic implications. This heterogeneity complicates patient management and underscores the urgent need for reliable prognostic biomarkers and tailored treatment strategies [16].

Higher levels of total tumor-infiltrating lymphocytes (TILs) generally indicate a favorable prognosis in breast cancer, but their prognostic value depends on the composition of immune cell subsets within the tumor microenvironment. Effector cells, such as CD8^+^ T cells, tissue-resident memory T cells, and natural killer (NK) cells, promote antitumor activity and are associated with improved clinical outcomes. These cells can be further enhanced through immunotherapies including PD-1/PD-L1 blockade, CAR T-cell approaches, and NK-cell activation strategies. Tumor-infiltrating B cells may also support antitumor immunity by producing cytotoxic antibodies and presenting antigens to activate T cells. In contrast, regulatory T cells (Tregs) and regulatory B cells (Bregs) exert immunosuppressive effects that inhibit cytotoxic responses, correlate with poorer prognosis, and represent important targets for therapeutic intervention. Overall, the prognostic significance of TILs reflects the balance between antitumor effector populations and immunosuppressive regulatory cells [17].

In parallel, research into the dynamic interactions between the immune system and cancer has underscored the critical role of the tumor immune microenvironment in influencing tumor progression and prognosis across multiple malignancies [17,18]. One of the key elements of this microenvironment is the presence of TILs, which are particularly abundant in TNBC, the subtype most frequently infiltrated by these immune cells [18,19]. Importantly, higher levels of TILs have been associated with increased rates of pathological complete response, as well as improved overall and disease-free survival in patients with various breast cancer subtypes [20–22].

TILs are composed of diverse lymphocyte populations, including cytotoxic CD8⁺ T cells, CD4⁺ helper T cells, B cells, and regulatory T cells (Tregs), each of which may exert distinct and sometimes opposing effects on tumor progression. For example, high CD8^+^ T-cell infiltration is generally associated with favorable prognosis, whereas increased Treg density has been linked to immunosuppression and poorer outcomes. These nuances highlight the need for a deeper understanding of how different immune cell subsets contribute to TNBC behavior [20].

International efforts have sought to standardize the evaluation of TILs in breast cancer. Notably, the International Immuno-Oncology Biomarker Working Group has developed guidelines for the assessment of stromal and intratumoral TILs to promote consistency and reproducibility in research and clinical practice. Such standardized criteria are essential for enabling meaningful comparisons across studies and for advancing TILs as potential biomarkers [21,22].

Although numerous studies have demonstrated associations between TIL levels and clinical outcomes in breast cancer, significant uncertainty remains regarding their prognostic relevance specifically within the context of TNBC. Existing systematic reviews often evaluate mixed breast cancer populations or focus broadly on immunological markers, making it unclear whether their conclusions can be reliably applied to TNBC, a biologically distinct and highly heterogeneous subtype. Moreover, the results reported across individual studies are not entirely consistent, with variations in TIL assessment methods, cutoffs, and the immune cell subsets analyzed contributing to conflicting findings. This lack of TNBC-focused, methodologically consistent evidence highlights the need for a dedicated systematic review to clarify the prognostic value of TILs in this subtype and to support more precise clinical application [23–25].

These findings highlight the potential of TIL density as a prognostic biomarker for TNBC, with implications for patient stratification and the development of more personalized treatment approaches. Therefore, this systematic review aims to assess the prognostic value of TILs in the survival outcomes of patients with TNBC.

## Materials and methods

### Study registration and reporting

This systematic review protocol was conducted following the methodological framework of the Preferred Reporting Items for Systematic Reviews and Meta-Analyses Protocols (PRISMA-P) [26] guidelines. The review has been registered in the International Prospective Register of Systematic Reviews (PROSPERO) under the protocol number: CRD42024568600.

Record screening was completed in August 2025, while data extraction is currently ongoing and expected to be finalized by December 2025. The analysis and preparation of results are projected to be completed by February 2026.

The review questions guiding this proposal are: What is the prognostic value of TILs in the survival outcomes of patients with TNBC? This question is justified by the persisting uncertainty and variability in literature regarding the magnitude and consistency of the association between TILs and TNBC survival.

### Eligibility criteria

**Types of study:** Cohort, case-control, and clinical trials will be included.

**Patients:** women with TNBC. The TNBC confirmed by immunohistochemical tests performed on a breast tissue sample obtained by biopsy.

**Exposure:** presence of TILs.

**Comparator/Control:** not applicable.

**Outcome measures:** The main outcome is overall survival of patients with TNBC. The secondary outcomes included correlation between disease-free survival, cancer-specific survival, and recurrence rate with the presence of TILs in TNBC.

### Exclusion criteria

Non-peer-reviewed articles, review articles, case reports, and case series will be excluded. Additionally, studies evaluating other types of breast cancer, insufficient data collected or those that fail to meet the predefined eligibility criteria will not be included.

### Search strategy

The following databases will be utilized in research: PubMed, Embase, Lilacs, Web of Science, Scopus, SciELO, ScienceDirect, and the Cochrane Library. Additionally, the reference lists of previously selected articles will be manually reviewed to identify potential eligible studies that may not have been captured in the initial search. No language or date restrictions will be applied during the selection process. The keywords used in the search will be based on Medical Subject Headings (MeSH), with the following combinations: “tumor-infiltrating lymphocytes,” “T lymphocytes,” “T cells,” “regulatory T cells,” “triple-negative breast neoplasm,” “triple-negative breast cancer,” “ER-negative PR-negative HER2-negative breast cancer,” “breast cancer,” and “triple-negative”. The search strategy for all databases is provided in supplementary file 1 available.

### Data and analysis

#### Study selection.

**Fig 1:**
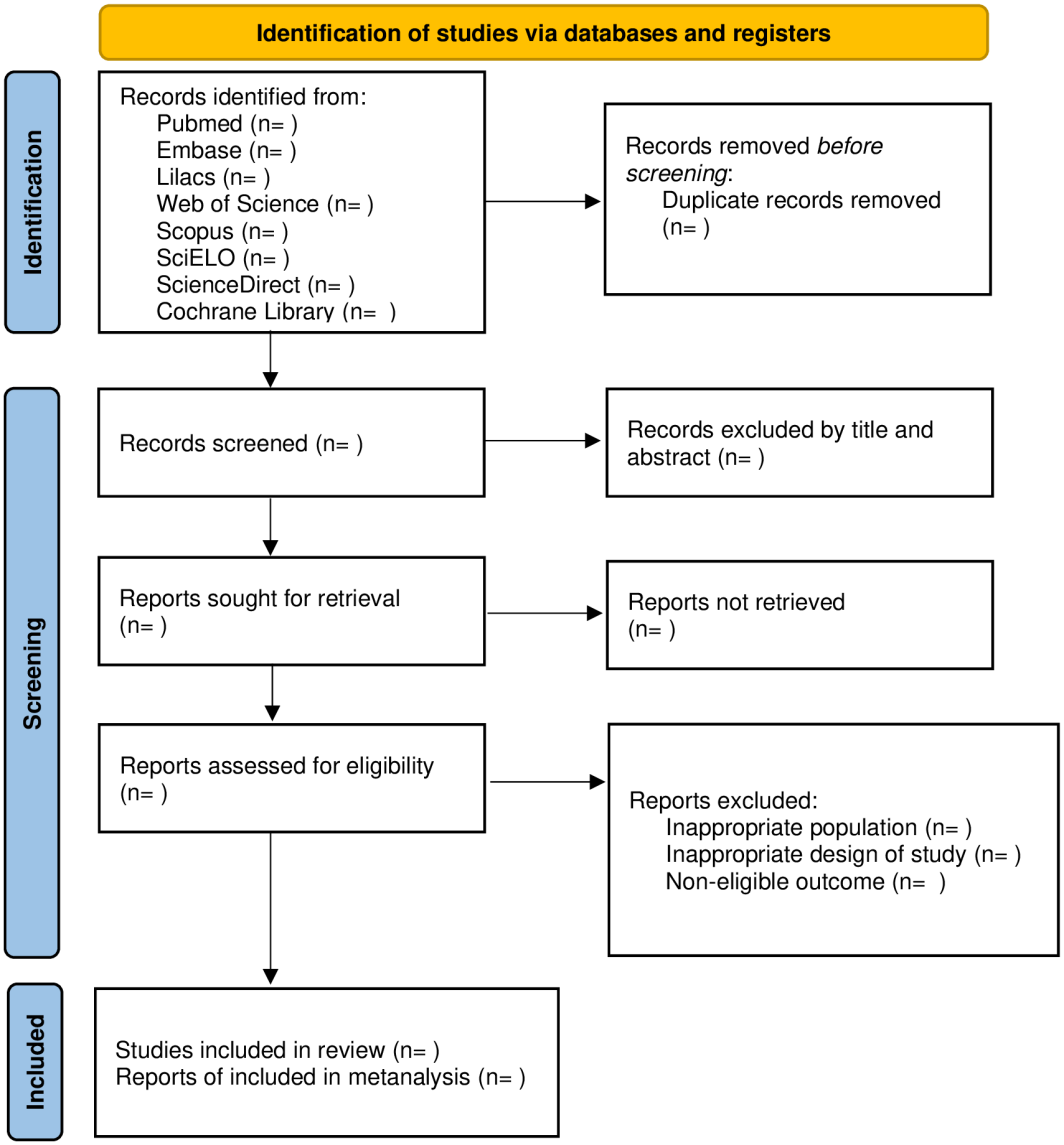
PRISMA flow diagram for systematic review and meta-analysis.

The analysis of the articles will be conducted using the Rayyan software. Initially, the duplicates will be removed. After, two authors (LCMA and EISA) will independently evaluate the outcomes, selecting studies based on their titles and abstracts. Disagreements regarding study selection will be resolved by a third reviewer (AKG), who will discuss and address any inconsistencies. The information on the phases of the selection process will be described through a Preferred Reporting Items for Systematic Reviews and Meta-Analyses Protocols diagram (PRISMA Flow diagram – Fig 1) [27].

### Data extraction

Following the selection of studies, two reviewers (LCMA and LMFN) will extract the research data. Disagreements regarding study selection will be resolved by a third reviewer (AKG). Customized forms have been developed by the authors to facilitate data collection and extraction. For each included study, the following information will be collected:

- Study Characteristics: Author, year of publication, country, study design, sample size, and detailed TIL-related methodological characteristics. These include the definition and method of TIL assessment (H&E stromal TILs; IHC subtypes such as CD8 + , FoxP3+), TIL phenotype, TIL concentration, TIL detection method, location of TILs, and the definition of high TIL levels. Information on TIL cut-off values used (e.g., > 10%, > 20%, > 50%).
- Patient Characteristics: Age, comorbidities when available, performance status (ECOG, Karnofsky, or study-specific).
- Disease Characteristics: Disease stage according to TNM or study-defined classification (early-stage, locally advanced, metastatic).
- Treatment Characteristics: Treatment setting: neoadjuvant, adjuvant, or systemic therapy for advanced disease, chemotherapy regimen: anthracycline-based, platinum-containing, taxanes, or combination regimens, and additional treatments (surgery type, radiotherapy).
- Outcomes: survival endpoints: overall survival (OS), disease-free survival (DFS), progression-free survival (PFS).

### Missing data

In cases of incomplete or missing data, the authors will attempt to contact the corresponding authors of the respective articles via telephone or email. If no response is obtained, the data will be excluded from the analysis, however, it will be addressed in the discussion section.

### Data synthesis

The meta package in R software (version 4.3.1) will be used to combine findings from clinically similar studies into a meta-analysis. Effect size metrics will include Hazard Ratios (HR) with 95% confidence intervals (CI) for time-to-event outcomes and Relative Risks (RR) for dichotomous outcomes. Heterogeneity across studies will be quantified using the I^2^ statistic. Given the expected methodological and clinical variability, a random-effects model will be employed. A two-sided p-value < 0.05 will be considered statistically significant.

To address heterogeneity in TILs assessment, this review will include studies evaluating stromal TILs by H&E, following the recommendations of the International TILs Working Group. Studies using immunohistochemical TIL subtypes (e.g., CD8 + , FoxP3+) will be analyzed separately and excluded from pooled H&E-based estimates. Differences in TIL cut-off values (e.g., > 10%, > 20%, > 50%) will be addressed through subgroup analyses and, when feasible, by synthesizing results using continuous TIL measures or preserving original study-defined categories.

Given that survival outcomes in TNBC may vary substantially by disease extent, disease stage (early-stage vs. locally advanced vs. metastatic) will be explicitly extracted and incorporated into the analysis. To prevent bias in overall survival (OS) estimates, studies including mixed stages will be handled through stratified subgroup analyses or excluded from specific pooled comparisons if stage data cannot be disaggregated.

Treatment-related characteristics will also be extracted and analyzed, including neoadjuvant vs. adjuvant therapy and chemotherapy regimen (anthracycline-based vs. platinum-containing regimens). Planned subgroup analyses will evaluate whether these treatment modalities modify the prognostic association between TILs and survival outcomes.

When sufficient data are available, meta-regression analyses will be conducted to explore whether variations in disease stage, treatment type, patient characteristics (age, comorbidities, performance status), or methodological factors contribute to between-study heterogeneity.

### Quality assessment

The Quality in Prognosis Studies (QUIPS) [28] tool will be independently applied by ACAS, and EMS to evaluate potential sources of bias in the identified prognostic studies. These biases relate to the following domains: (1) study participation, (2) study attrition, (3) measurement of the prognostic factor, (4) outcome measurement, (5) study confounding, and (6) statistical analysis and reporting. If more than ten studies meet the eligibility criteria, publication bias will be evaluated using Egger’s and Begg’s regression models, alongside a visual inspection of the funnel plot for asymmetry. Disagreements regarding study selection will be resolved by a third reviewer (KSM).

### Patient and public involvement

Patients and/or the public were not involved in the design, performance, reporting, or dissemination plans of this research. Patient consent for publication is not applicable.

### Ethical principles

The findings of this study will be disseminated through publication in a peer-reviewed open-access scientific journal, as well as through additional scientific publications and reports. Ethical review and approval are not required, as the analysis will reach only data obtained from open access literature.

## Discussion

Triple-negative breast cancer (TNBC) is the breast cancer subtype associated with the poorest prognosis, primarily affecting younger women, particularly those under 40 years of age. This subtype is marked by an aggressive clinical course, rapid progression, and a high risk of recurrence and metastasis [1,29].

Recent studies have highlighted the prognostic relevance of tumor-infiltrating lymphocytes (TILs), especially those located in the tumor stroma. Evidence suggests a significant positive correlation between high TIL density and improved survival outcomes in patients with TNBC [30]. TILs have emerged as promising, cost-effective biomarkers that can be integrated into routine clinical practice for both early and advanced stages of the disease [17,31]. When assessed in biopsy samples, a TIL density greater than 10% is associated with a reduced risk of recurrence compared to densities below 10% [32]. Furthermore, higher TIL levels are consistently linked to improved invasive disease-free survival, distant disease-free survival, and overall survival [33].

Given TNBC's highly immunogenic nature, driven by a tumor microenvironment rich in TILs, immunotherapy has become an increasingly promising treatment avenue [34]. Modulating the tumor microenvironment to enhance TIL infiltration could further boost immunotherapeutic efficacy [35]. Additionally, the expression of Programmed Cell Death-Ligand 1 (PD-L1), a key immunotherapy target, is strongly associated with TIL presence, reinforcing their central role in the development of novel therapeutic strategies for TNBC [36–40].

Despite advances in understanding the disease, TNBC remains heterogeneous, and its current gold-standard treatment continues to be chemotherapy. Common agents include anthracyclines, alkylating agents, taxanes, and 5-fluorouracil, often administered as neoadjuvant therapy followed by surgery in early-stage cases. However, for refractory or advanced disease, there is no universally accepted standard of care, and responses to treatment are typically short-lived, frequently followed by metastatic recurrence [26,27]. As of now, the primary prognostic measure in clinical settings remains the anatomical tumor burden [28].

### Limitations

This systematic review and meta-analysis may face some limitations. First, significant heterogeneity is expected among the included studies due to differences in methods used to assess TILs, such as staining techniques, quantification criteria, and the specific regions of the tumor evaluated. Second, despite the existence of international guidelines, the lack of standardization in TIL quantification across studies can compromise comparability and consistency of the findings. Third, incomplete or inconsistently reported data, such as missing hazard ratio values, TIL concentrations, or patient characteristics—may limit the inclusion of studies or lead to the exclusion of relevant data from the final analysis. Lastly, the observational nature of most included studies makes them inherently susceptible to biases related to selection and information. These limitations should be considered when interpreting the results of this review**.**

## Supporting information

S1 File Suplementary file 1. (DOCX)

S1 Checklist WPRISMA-P 2015. (DOCX)
