# Supplementary material for: Analysis of tumor-infiltrating lymphocytes as prognostic factor in triple-negative breast cancer: A protocol of systematic review and meta-analysis
Source: PLoS One. 2026 May 12;21(5):e0342085. doi: 10.1371/journal.pone.0342085 (PMC13166935; doi:10.1371/journal.pone.0342085)
Supplement: S1 File — (DOCX) [file pone.0342085.s001.docx]

**Supplementary file 1**. Search strategy for all databases.

| **Database** | **Search strategy** | **Number of articles** | **Date** |
| --- | --- | --- | --- |
| EMBASE | ('triple negative breast cancer'/exp OR 'tnbc' OR 'metastatic triple-negative breast cancer' OR 'triple negative breast cancer' OR 'triple negative breast cancers' OR 'triple negative breast carcinoma' OR 'triple negative breast carcinomas' OR 'triple negative breast neoplasm' OR 'triple negative breast neoplasms' OR 'triple receptor negative breast cancer' OR 'triple receptor negative breast cancers' OR 'triple-negative bc' OR 'triple-negative metastatic breast cancer' OR 'triple-negative subset of breast cancer') AND ('tumor associated leukocyte'/exp OR 'cancer associated leucocyte' OR 'cancer associated leukocyte' OR 'cancer associated lymphocyte' OR 'intratumoral leukocyte' OR 'intratumoral lymphocyte' OR 'intratumoural lymphocyte' OR 'lymphocytes, tumor-infiltrating' OR 'lymphocytes, tumour-infiltrating' OR 'tumor associated leukocyte' OR 'tumor associated lymphocyte' OR 'tumor infiltrating leukocyte' OR 'tumor infiltrating lymphocyte' OR 'tumor-infiltrating lymphocytes' OR 'tumour associated leucocyte' OR 'tumour associated lymphocyte' OR 'tumour infiltrating leucocyte' OR 'tumour infiltrating lymphocyte' OR 'tumour-infiltrating lymphocytes') AND ('overall survival'/exp OR 'overall survival' OR 'recurrence risk'/exp OR 'recidivation risk' OR 'recidivism risk' OR 'recurrence rate' OR 'recurrence risk' OR 'relapse rate' OR 'risk recidivism' OR 'risk, recurrence' OR 'disease free survival'/exp OR 'dfs (disease free survival)' OR 'disease free survival' OR 'disease-free survival' OR 'cancer specific survival'/exp OR 'cancer specific survival' OR 'leukemia free survival' OR 'leukemia survival') | 1251 | 01/20/2025 |
| COCHRANE | (("Triple Negative Breast Neoplasms” OR "ER-Negative PR-Negative HER2-Negative Breast Cancer" OR "ER Negative PR Negative HER2 Negative Breast Cancer" OR "ER-Negative PR-Negative HER2-Negative Breast Neoplasms" OR "ER Negative PR Negative HER2 Negative Breast Neoplasms" OR "Triple Negative Breast Cancer" OR "Triple-Negative Breast Cancer" OR "Breast Cancers, Triple-Negative" OR "Breast Cancer, Triple-Negative" OR "Triple-Negative Breast Cancers" OR "Triple-Negative Breast Neoplasm" OR "Breast Neoplasms, Triple-Negative" OR "Breast Neoplasm, Triple-Negative" OR "Triple Negative Breast Neoplasm" OR "Triple-Negative Breast Neoplasms") AND ("lymphocytes, tumor infiltrating” OR "Tumor-Infiltrating Lymphocyte" OR "Lymphocyte, Tumor-Infiltrating" OR "Tumor-Infiltrating Lymphocytes" OR "Tumor Infiltrating Lymphocyte" OR "Infiltrating Lymphocytes, Tumor" OR "Infiltrating Lymphocyte, Tumor" OR "Lymphocytes, Tumor Infiltrating" OR "Lymphocyte, Tumor Infiltrating" OR "Tumor Infiltrating Lymphocytes" OR "Tumor-Derived Activated Cells" OR "Activated Cells, Tumor-Derived" OR "Activated Cell, Tumor-Derived" OR "Tumor Derived Activated Cells" OR "Tumor-Derived Activated Cell" OR "Tumor Derived Activated Cell") AND ("Recurrence” OR "Recurrences" OR "Relapse" OR "Relapses" OR "Recrudescence" OR "Recrudescences" OR "Neoplasm Recurrence, Locoregional" OR "Locoregional Neoplasm Recurrences" OR "Neoplasm Recurrences, Locoregional" OR "Recurrences, Locoregional Neoplasm" OR "Neoplasm Recurrences, Local" OR "Recurrence, Local Neoplasm" OR "Recurrence, Locoregional Neoplasm" OR "Recurrences, Local Neoplasm" OR "Local Neoplasm Recurrence" OR "Locoregional Neoplasm Recurrence" OR "Local Neoplasm Recurrences" OR "Overall" OR "Disease-Free Survival" OR "Disease Free Survival" OR "Survival, Disease-Free" OR "Survival, Disease Free")) in Title Abstract Keyword | 85 |  |
| LILACS | (Triple Negative Breast Neoplasms OR Triple Negative Breast Cancer OR Triple-Negative Breast Cancer OR Breast Cancers, Triple-Negative OR Breast Cancer, Triple-Negative OR Triple-Negative Breast Cancers OR Triple-Negative Breast Neoplasm OR Breast Neoplasms, Triple-Negative OR Breast Neoplasm, Triple-Negative OR Triple Negative Breast Neoplasm OR Triple-Negative Breast Neoplasms) AND (lymphocytes, tumor infiltrating OR Tumor-Infiltrating Lymphocyte OR Lymphocyte, Tumor-Infiltrating OR Tumor-Infiltrating Lymphocytes OR Tumor Infiltrating Lymphocyte OR Infiltrating Lymphocytes, Tumor OR Infiltrating Lymphocyte, Tumor OR Lymphocytes, Tumor Infiltrating OR Lymphocyte, Tumor Infiltrating OR Tumor Infiltrating Lymphocytes OR Tumor-Derived Activated Cells OR Activated Cells, Tumor-Derived OR Activated Cell, Tumor-Derived OR Tumor Derived Activated Cells OR Tumor-Derived Activated Cell OR Tumor Derived Activated Cell) AND (Recurrence OR Recurrences OR Relapse OR Relapses OR Recrudescence OR Recrudescences OR Disease Free Survival OR Survival, Disease-Free OR Survival, Disease Free OR Disease-Free Survival OR Overall) | 5 | 01/20/2025 |
| Pubmed | (("Triple Negative Breast Neoplasms” OR "ER-Negative PR-Negative HER2-Negative Breast Cancer" OR "ER Negative PR Negative HER2 Negative Breast Cancer" OR "ER-Negative PR-Negative HER2-Negative Breast Neoplasms" OR "ER Negative PR Negative HER2 Negative Breast Neoplasms" OR "Triple Negative Breast Cancer" OR "Triple-Negative Breast Cancer" OR "Breast Cancers, Triple-Negative" OR "Breast Cancer, Triple-Negative" OR "Triple-Negative Breast Cancers" OR "Triple-Negative Breast Neoplasm" OR "Breast Neoplasms, Triple-Negative" OR "Breast Neoplasm, Triple-Negative" OR "Triple Negative Breast Neoplasm" OR "Triple-Negative Breast Neoplasms") AND ("lymphocytes, tumor infiltrating” OR "Tumor-Infiltrating Lymphocyte" OR "Lymphocyte, Tumor-Infiltrating" OR "Tumor-Infiltrating Lymphocytes" OR "Tumor Infiltrating Lymphocyte" OR "Infiltrating Lymphocytes, Tumor" OR "Infiltrating Lymphocyte, Tumor" OR "Lymphocytes, Tumor Infiltrating" OR "Lymphocyte, Tumor Infiltrating" OR "Tumor Infiltrating Lymphocytes" OR "Tumor-Derived Activated Cells" OR "Activated Cells, Tumor-Derived" OR "Activated Cell, Tumor-Derived" OR "Tumor Derived Activated Cells" OR "Tumor-Derived Activated Cell" OR "Tumor Derived Activated Cell") AND ("Recurrence” OR "Recurrences" OR "Relapse" OR "Relapses" OR "Recrudescence" OR "Recrudescences" OR "Neoplasm Recurrence, Locoregional" OR "Locoregional Neoplasm Recurrences" OR "Neoplasm Recurrences, Locoregional" OR "Recurrences, Locoregional Neoplasm" OR "Neoplasm Recurrences, Local" OR "Recurrence, Local Neoplasm" OR "Recurrence, Locoregional Neoplasm" OR "Recurrences, Local Neoplasm" OR "Local Neoplasm Recurrence" OR "Locoregional Neoplasm Recurrence" OR "Local Neoplasm Recurrences" OR "Overall" OR "Disease-Free Survival" OR "Disease Free Survival" OR "Survival, Disease-Free" OR "Survival, Disease Free")) | 350 | 01/20/2025 |
| Science direct | ("triple negative breast cancer") AND ("tumor infiltrating lymphocytes") AND (recurrence OR "disease free survival") | 1.490 | 01/20/2025 |
| Scopus | ( TITLE-ABS-KEY ( "triple negative breast neoplasms" ) OR TITLE-ABS-KEY ( "triple negative breast cancer" ) AND TITLE-ABS-KEY ( "tumor-infiltrating lymphocyte" ) OR TITLE-ABS-KEY ( "lymphocytes, tumor infiltrating" ) OR TITLE-ABS-KEY ( "tumor-derived activated cells" ) AND TITLE-ABS-KEY ( recurrence ) OR TITLE-ABS-KEY ( "neoplasm recurrence, local" ) OR TITLE-ABS-KEY ( "disease-free survival" ) OR TITLE-ABS-KEY ( overall ) )TITLE-ABS-KEY (("Triple Negative Breast Neoplasms") AND ("Tumor-Infiltrating Lymphocyte" OR "lymphocytes, tumor infiltrating" OR "Tumor-Derived Activated Cells") AND ("Recurrence" OR "neoplasm recurrence, local" OR "Disease-Free Survival" OR "Overall") | 437 | 01/20/2025 |
| Scielo | (Triple Negative Breast Neoplasms OR Triple Negative Breast Cancer) AND (Tumor Infiltrating Lymphocyte)) AND (Overall OR Recurrence) | 0 | 01/20/2025 |
| Web of science | ("Triple Negative Breast Neoplasms” OR "Triple Negative Breast Cancer" OR "Triple-Negative Breast Cancer" OR "Triple-Negative Breast Cancers" OR "Triple-Negative Breast Neoplasm" OR "Breast Neoplasms, Triple-Negative" OR "Breast Neoplasm, Triple-Negative" OR "Triple Negative Breast Neoplasm") AND ("lymphocytes, tumor infiltrating” OR "Tumor-Infiltrating Lymphocyte" OR "Tumor-Infiltrating Lymphocytes" OR "Tumor Infiltrating Lymphocyte" OR "Infiltrating Lymphocytes, Tumor" OR "Lymphocyte, Tumor Infiltrating") AND ("Recurrence” OR "Relapse" OR "Recrudescence" OR "Neoplasm Recurrence, Locoregional" OR "Neoplasm Recurrences, Locoregional" OR "Recurrences, Locoregional Neoplasm" OR "Neoplasm Recurrences, Local" OR "Recurrence, Locoregional Neoplasm" OR "Local Neoplasm Recurrence" OR "Locoregional Neoplasm Recurrence" OR "Overall" OR "Disease Free Survival" OR "Survival, Disease-Free") | 476 | 01/20/2025 |
